# Tidyplots empowers life scientists with easy code‐based data visualization

**DOI:** 10.1002/imt2.70018

**Published:** 2025-03-19

**Authors:** Jan Broder Engler

**Affiliations:** ^1^ Institut für Neuroimmunologie und Multiple Sklerose Zentrum für Molekulare Neurobiologie Hamburg, Universitätsklinikum Hamburg‐Eppendorf Hamburg Germany

**Keywords:** data analysis, data science, data visualization, R package, tidyverse

## Abstract

Code‐based data visualization is a crucial tool for understanding and communicating experimental findings while ensuring scalability and reproducibility. However, complex programming interfaces pose a significant barrier for life scientists. To address this challenge, tidyplots provides a user‐friendly code‐based interface for creating customizable and insightful plots. With its consistent and intuitive syntax, tidyplots empowers researchers to leverage automated data visualization pipelines while minimizing required programming skills.

## Highlights

- Easy code‐based data visualization for the life sciences.
- Intuitive syntax to add, remove, and adjust plot components.
- Reduced code complexity in comparison to ggplot2.
- Includes built‐in demo datasets, color schemes, and a beginner‐friendly getting started guide.

## Graphical abstract

Effective data visualization is vital for analyzing and communicating experimental results. However, most code‐based plotting tools require substantial programming experience, posing a significant barrier to entry for life scientists. Tidyplots makes code‐based plotting more accessible by offering an intuitive interface for generating highly customizable and informative plots.

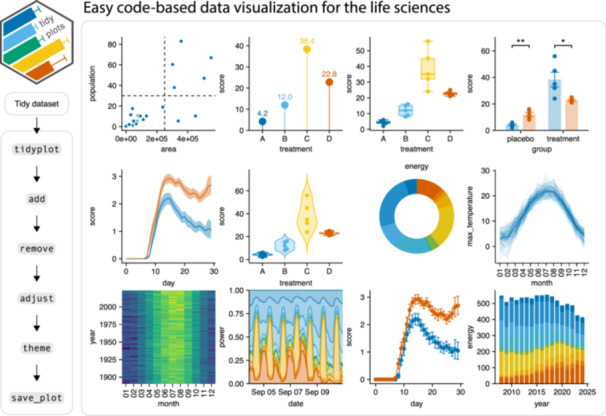

## INTRODUCTION

Data visualization is a crucial component of the data analysis workflow, facilitating efficient data exploration and the extraction of experimental findings, including their direction, magnitude, and robustness. Additionally, it is a key tool for communicating findings in scientific outlets and engaging the community in discussions to validate, corroborate, or challenge results [1, 2].

Advances in life sciences methodology have led to a surge in both the volume and complexity of experimental data. As a result, traditional data analysis workflows—often reliant on copy‐pasting and manual spreadsheet manipulations—are struggling to meet the demands for higher throughput and the rising standards of reproducibility and transparency. This has prompted the life science community to adopt programmatic data analysis ecosystems. The most widely used tools include R‐based packages like the tidyverse [3] and ggplot2 [4], and Python‐based libraries such as Pandas, NumPy, Matplotlib, and Seaborn.

However, despite their utility and power, each of these tools uses a specialized syntax and requires significant coding experience, posing a barrier to adoption by life scientists. Something as simple as a scatter plot with error bars and a statistical test typically requires a considerable amount of code and intricate knowledge of the plotting tool's internal workings. To address this challenge, this paper introduces the open‐source R package “tidyplots”, which was specifically designed to empower life scientists to benefit from automated data visualization pipelines.

## RESULTS

Tidyplots is based on ggplot2 and was devised to address similar needs as ggstatsplot [5] and ggpubr [6]; however, instead of extending the ggplot2 syntax, tidyplots introduces a novel interface based on a consistent and intuitive grammar that minimizes the need of programming experience.

**Figure 1:**
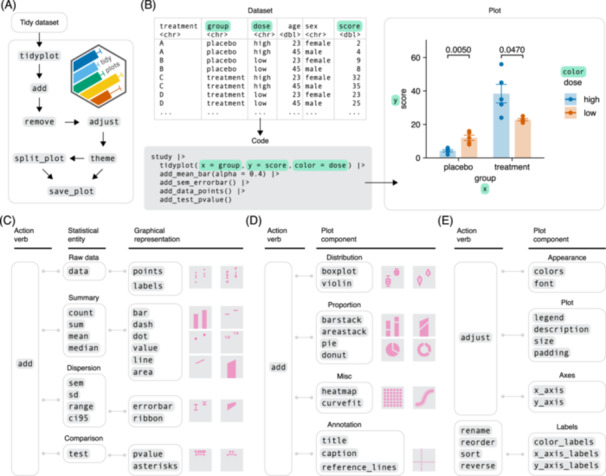
Design principles of tidyplots. (A) Overview of the tidyplots workflow. (B) Scheme and example code illustrating the way from the data set to the plot. (C) Grammar of add functions showing available combinations of statistical entities and graphical representations. (D) Grammar of add functions for predefined plot components. (E) Grammar of functions to adjust the plot.

The tidyplots workflow is composed of a series of function calls that are connected in one pipeline (Figure 1A). After starting the plot using the tidyplot() function, there are three main verbs to construct and modify the plot, namely, add, remove, and adjust. Optionally, the user can choose a different theme, split the plot into a multiplot layout, or save it to file (Figure 1A). As an example, we will use the *study* data set from the tidyplots package that includes a treatment and placebo group at two different treatment doses and a score measuring treatment success (Figure 1B). Within the tidyplot() function, we define which variable to use for the *x*‐axis, the *y*‐axis, and the color. In the next line, we add the mean value of each group represented as a bar using the function add_mean_bar(). This exemplifies a general scheme, in which function names start with an action verb, such as add, followed by the statistical entity, such as mean, followed by the graphical representation of the entity, such as bar. In a similar way, we can add the standard error of the mean as error bar with add_sem_errorbar(), the raw data values as points with add_data_points(), and a statistical test as *p* values with add_test_pvalue() (Figure 1B). Tidyplots comes with over 50 add functions that cover the plotting of raw data values, summary statistics, dispersion, distribution, proportion, annotation and statistical comparison (Figure 1C,D).

Fortunately, the user does not have to memorize all available function names, because auto‐completion in current code editors readily suggests fitting functions after typing a few characters. Thus, typing add_ gives a list of all add functions, typing add_mean gives a list of all available graphical representations of mean, and typing add_bar gives a list of all available statistical entities that can be represented as bars.

In the next step, the user can remove unwanted plot elements and adjust the appearance of the plot, including colors, fonts, legend, titles, size, padding, and axes (Figure 1E). A specialized group of functions deals with the color and axis labels that are derived from the data set itself. Starting with rename, reorder, sort and reverse, these functions allow to conveniently modify the order and naming of data labels along the axes and the color legend (Figure 1E).

**Figure 2:**
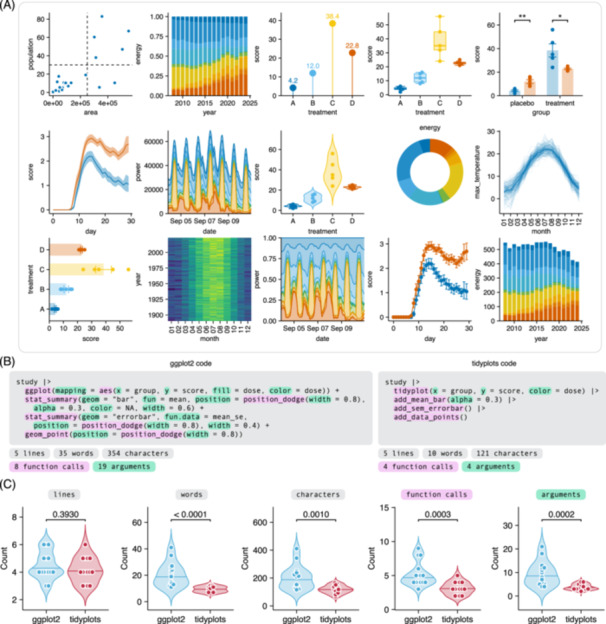
Performance of tidyplots. (A) Plot gallery. (B) ggplot2 and tidyplots example code to generate equivalent plots. (C) Comparison of ggplot2 and tidyplots code across plots in (A). Assessed metrics include the number of lines, words, characters, function calls, and arguments in the ggplot2 code versus tidyplots code. Statistical analysis was performed by the Mann–Whitney *U* test.

Given its modular architecture, tidyplots can be used to create a wide range of scientific plots (Figure 2A), while its focus on human code readability makes the underlying source code easy to read and write. To illustrate this, we will compare tidyplots to the most popular R plotting package ggplot2. Due to its more granular interface the ggplot2 code is less accessible and requires a deeper understanding of several internal ggplot2 concepts (Figure 2B). In contrast, tidyplots requires considerably less words, characters, function calls, and function arguments to achieve plots equivalent to Figure 2A, thus demonstrating a significant reduction of code complexity (Figure 2C).

**Figure 3:**
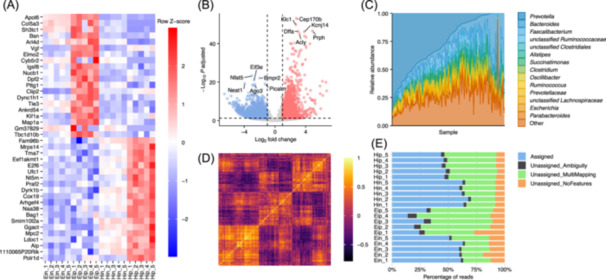
Tidyplots use cases. (A) Gene expression heatmap. (B) Volcano plot. (C) Microbiome composition. (D) Gene correlation heatmap. (E) RNA‐seq quality control. Data adapted from Schattling et al. [7] in (A), (B), and (E). Tamburini et al. [8] in (C). Spellman et al. [9] in (D).

By embracing the ggplot2 concept of incrementally adding layers of information, tidyplots maintains a high degree of flexibility, enabling the creation of plots tailored to diverse data visualization needs and use cases. These include common plot types in biostatistics and bioinformatics (Figure 3A–E), but also in social sciences, natural sciences, and data journalism. A continuously evolving collection of such use cases together with the required data and code can be found at https://tidyplots.org/use-cases/.

Because tidyplots is based on ggplot2, it allows seamless integration of ggplot2 code. Thereby, tidyplots provides access to a wide range of features that are implemented within ggplot2 or ggplot2 extension packages. To streamline this integration, tidyplots provides the add() helper function, enabling the addition of ggplot2 code without disrupting the tidyplots pipeline. However, there are also notable design differences between tidyplots and ggplot2. One key distinction is tidyplots' consistent use of absolute dimensions. By default, a newly created tidyplot has a plot area size of 50 by 50 mm. These dimensions can be easily adjusted using the adjust_size() function. This approach ensures that the proportions of plot area size, font size, plot elements, and line strength remain consistent. Consequently, a tidyplot retains its size and composition independent of the size of the graphics device or display window. To save tidyplots to file, the save_plot() function automatically extracts the plot's absolute dimensions to determine the appropriate size for the graphics device used to write the output file.

## CONCLUSION

Overall, tidyplots provides a powerful and user‐friendly solution for creating code‐based scientific plots. Beyond its core functionality, tidyplots offers several valuable features, including demo datasets that are ideal for learning and teaching, color schemes optimized for individuals with color vision deficiencies, and thorough documentation with a beginner‐friendly getting started guide. Tidyplots also enhances the productivity of experienced users by streamlining the process of creating complex visualizations, allowing them to focus more on data interpretation and analysis. By promoting the use of code‐based plotting in the life sciences, tidyplots has the potential to accelerate scientific discoveries and enhance reproducibility and transparency.

## METHODS

### Interface design

The interface of tidyplots is heavily inspired by the tidyverse ecosystem [3] and its underlying design principles [10]. Most importantly, tidyplots is designed (i) to maximize human code readability by using natural language elements, (ii) to be consistent across functions and reduce the need to remember special cases, and (iii) to be modular in the sense that a complex task can be broken down into multiple steps. Function names begin with an action verb, such as add, remove, adjust, or save, clearly indicating the type of action the function performs. For adding summary statistics, statistical entities are given precedence over graphical presentation, encouraging a conscious decision about *what* to plot, such as mean, before *how* to represent it, such as bar.

Similar to ggplot2 [4], which is the plotting framework underlying tidyplots, plots are built by gradually adding layers of information. This modular design enables maximal flexibility and freedom to combine elements to compose complex plots. However, a main difference between ggplot2 and tidyplots is that ggplot2 functions represent nouns that are added together using the + operator while tidyplots functions represent verbs that are combined in a pipeline using the pipe operator. The concept of piping has been widely adopted in R programming because it greatly enhances code readability. By using the pipe, tidyplots aims to increase the consistency across data analysis workflows. Thus, the wrangling of data sets, the plotting, and the post‐processing of the plots, such as multiplot arrangement and saving, can be conveniently combined in one pipeline without the need to switch operators.

Another design decision of tidyplots concerns the prioritization of specialized functions over function arguments. For example, the addition of the mean in several graphical representations could be implemented by one function called add_mean() that takes the desired graphical representation, such as bar, dash, or point, as parameter. Instead, tidyplots implements three individual functions called add_mean_bar(), add_mean_dash() and add_mean_point(). Thereby, tidyplots encouraged the use of autocompletion in the code editor that gives a list of all available options while typing, thus eliminating the need to consult function documentation. In fact, most tidyplot functions work as intended without any parameters, greatly accelerating the writing of code, improving code readability, and facilitating the learning of tidyplots. Following this notion, tidyplots offers over 50 add function, that adhere to the same consistent naming scheme.

### Functional programming

The above‐mentioned prioritization of specialized functions over function arguments comes with one central challenge in package development, which is code repetition. For example, add_mean_bar(), add_median_bar(), and add_sum_bar() deliver very similar functionality only differing in the statistical summary function they apply. When defining these functions, this would lead to considerable code repetition, which inflates the amount of source code and, more importantly, compromises code maintainability. To address this challenge, tidyplots makes heavy use of function factories [11]. Function factories are functions that return manufactured functions. For example, the tidyplots function factory ff_bar() takes a statistical summary function, such as mean, median, or sum, as input and delivers the manufactured functions add_mean_bar(), add_median_bar(), and add_sum_bar() as output. This approach eliminates code repetition and greatly enhances code maintainability.

### Dependencies

Tidyplots heavily relies on the ecosystem of tidyverse packages [3], especially ggplot2 [4]. However, to deliver specific features, tidyplots also depends on some additional great open‐source R packages. These include patchwork [12] to enable absolute plot dimensions, ggrastr [13] to enable rasterization of layers with too many vector shapes, ggbeeswarm [14] to avoid over‐plotting by violin‐shaped distribution of data points, ggrepel [15] to handle overlapping text labels, and ggpubr [6] for adding statistical comparisons directly to the plot.

### Benchmarking

For benchmarking code complexity, the code to generate the tidyplots plot gallery (Figure 2A) was analyzed using the tidyverse package stringr and compared to the ggplot2 code needed to generate equivalent plots. Benchmarking metrics included the number of lines, words, characters, functions calls, and function arguments in the ggplot2 code versus tidyplots code. The source code for the analysis along with the generated plots is available at https://github.com/jbengler/tidyplots_paper. Statistical analyses of benchmarking metrics were performed using Mann–Whitney *U* test.

## AUTHOR CONTRIBUTIONS

**Jan Broder Engler**: Conceptualization; funding acquisition; writing—original draft; writing—review and editing; visualization; validation; project administration; methodology; software; formal analysis; data curation; resources; investigation.

## CONFLICT OF INTEREST STATEMENT

The authors declare no conflicts of interest.

## ETHICS STATEMENT

No animals or humans were involved in this study.

## Data Availability

The data that support the findings of this study are openly available in tidyplots at https://github.com/jbengler/tidyplots/. Tidyplots is available on CRAN at https://CRAN.R-project.org/package=tidyplots and GitHub at https://github.com/jbengler/tidyplots. Documentation, articles and a getting started guide are available at https://tidyplots.org. The tidyplots source code is available at https://github.com/jbengler/tidyplots. The source code for Figure 2 and Figure 3 is available at https://github.com/jbengler/tidyplots_paper. All data bundled within the tidyplots package are available at https://github.com/jbengler/tidyplots. Data to reproduce tidyplots use cases are available at https://tidyplots.org/use-cases/. Supplementary materials (graphical abstract, slides, videos, Chinese translated version, and update materials) may be found in the online DOI or iMeta Science http://www.imeta.science/.
